# Development and validation of the short-form adolescent health promotion scale

**DOI:** 10.1186/1471-2458-14-1106

**Published:** 2014-10-26

**Authors:** Mei-Yen Chen, Li-Ju Lai, Hsiu-Chih Chen, Jorge Gaete

**Affiliations:** College of Nursing, Chang Gung University of Science and Technology, No. 2, Chiapu Rd. West Sec, Putz City, Chiayi County 61363 Taiwan; Department of Ophthalmology, Chang Gang Memorial Hospital, Putz City, Chiayi County 61363 Taiwan; School Health Nurse, Du-Pa Elementary School, Tainan, Taiwan; School of Psychology, Universidad de los Andes, Santiago, Chile

**Keywords:** Instrument development, Adolescent, Health promotion, Adolescent health promotion scale

## Abstract

**Background:**

Health-promoting lifestyle choices of adolescents are closely related to current and subsequent health status. However, parsimonious yet reliable and valid screening tools are scarce. The original 40-item adolescent health promotion (AHP) scale was developed by our research team and has been applied to measure adolescent health-promoting behaviors worldwide. The aim of our study was to examine the psychometric properties of a newly developed short-form version of the AHP (AHP-SF) including tests of its reliability and validity.

**Methods:**

The study was conducted in nine middle and high schools in southern Taiwan. Participants were 814 adolescents randomly divided into two subgroups with equal size and homogeneity of baseline characteristics. The first subsample (calibration sample) was used to modify and shorten the factorial model while the second subsample (validation sample) was utilized to validate the result obtained from the first one. The psychometric testing of the AHP-SF included internal reliability of McDonald’s omega and Cronbach's alpha, convergent validity, discriminant validity, and construct validity with confirmatory factor analysis (CFA).

**Results:**

The results of the CFA supported a six-factor model and 21 items were retained in the AHP-SF with acceptable model fit. For the discriminant validity test, results indicated that adolescents with lower AHP-SF scores were more likely to be overweight or obese, skip breakfast, and spend more time watching TV and playing computer games. The AHP-SF also showed excellent internal consistency with a McDonald’s omega of 0.904 (Cronbach’s alpha 0.905) in the calibration group.

**Conclusion:**

The current findings suggest that the AHP-SF is a valid and reliable instrument for the evaluation of adolescent health-promoting behaviors. Primary health care providers and clinicians can use the AHP-SF to assess these behaviors and evaluate the outcome of health promotion programs in the adolescent population.

**Electronic supplementary material:**

The online version of this article (doi:10.1186/1471-2458-14-1106) contains supplementary material, which is available to authorized users.

## Background

Health promotion is defined as a process of enabling people to increase control over and improve their health. This definition moves beyond a focus on individual behavior toward a wide range of potential social and environmental interventions [1]. Numerous studies and accumulating evidence show that practicing health promotion behaviors decreases the occurrence of disease and lowers death rate [2, 3]. Consequently, many societies focus on health promotion by identifying health improvement as an investment and promoting lifestyle changes.

In adulthood, good health-promoting behaviors depend on living habits adopted during early development [4]. The study of adolescent health-promoting behaviors is thus important, especially because adolescents are at a dynamic transition period bridging childhood to adulthood. This transition is characterized by rapid, interrelated changes in body, mind, and social relationships [4]. Most discussions concerning adolescent health promotion include the topic of improved bio-psychosocial wellbeing, for example, by enhancing regular exercise, nutrition, stress management, spiritual life, and interpersonal relationship behaviors [5, 6].

Although adolescents are often believed to be in the prime of health, adolescent behavior patterns can change rapidly and associated health issues may include irregular meals and sleep patterns, inactivity, poor eating habits, alcohol and tobacco use, reproductive health issues, illicit drug use, motor vehicle fatalities, homicide and violent crimes, unplanned pregnancy, chronic illness, and suicide [1, 7]. Research concerning adolescent health promotion issues has increased markedly since the 1980s and has been extended to include individual, family, and community contexts [6]. Such studies have shown that the prevalence of metabolic syndrome is high among adolescents who are inactive and adopt an inadequate diet; further, many countries report low levels of cardio-respiratory fitness in adolescents [8–11]. Some researchers have suggested that prevention strategies for obesity and metabolic syndrome should concentrate on enhancing fitness levels and making adequate dietary choices early in life [10, 12].

Numerous studies have demonstrated a strong association between sedentary life patterns (i.e., reduced physical activity and spending too much time watching television, sitting in front of the computer screen, or playing computer games) and health in youth [8, 9, 13]. There is also an abundance of literature showing that adolescents who perform regular physical activity experience lower levels of morbidity and mortality, even after adjustment for comorbid illnesses [6, 14, 15]. Further, a Swedish longitudinal cohort study demonstrated that increased adolescent muscle strength is a predictor of decreasing numbers of cardiovascular disease events and reduced mortality in middle age [16]. However, in real situations, clinicians or adolescent health care providers appear not to provide adequate health promotion counseling (e.g., promotion of exercise and healthy diet) because of limitations in available time, measurement tools, or related knowledge and skills [17].

Studies concerned with improving adolescent health could benefit from appropriate standardized instruments. During the past 10 years, Chen and colleagues [18, 19] have developed and validated a 40-item Adolescent Health Promotion (AHP-40) scale with the goal of measuring adolescent health in Taiwan. The AHP-40 assesses nutritional behaviors, social support, life appreciation, health responsibility, stress management, and exercise behaviors. To date, the scale has been used in more than 15 countries and there are currently five language versions. The scale has been applied by 212 researchers in the professional fields of nursing, school health, clinical pediatrics, dentistry, and mental health [20, 21]. Although the AHP-40 is reliable and valid, there are some disadvantages encountered in its application. One problem is that the scale is time-consuming to complete, which limits its application in clinical practice or study situations. Another problem is that experts (across countries and cultures) report that some AHP-40 items are redundant based on the results of exploratory factor analysis (EFA). Specifically, these experts report redundancies in the following subscales: (1) nutrition (“I eat three meals daily” and “I eat breakfast daily”), (2) health responsibility (“I read food labels when I shop” and “I make an effort to choose foods without preservatives”), and (3) life appreciation (“I make an effort to like myself” and “I make an effort to feel happy and content”). Based on these observations and reports, we concluded that the AHP-40 should be modified. Therefore, the aim of the present study was to examine the psychometric properties of a newly developed short-form version of the AHP (AHP-SF). We also tested the reliability and validity of this new instrument.

## Methods

### Participants and design

The present study formed part of a 2011 health promotion program for adolescents and was conducted in collaboration with the Bureau of Education in Tainan County, southern Taiwan. Data were collected using a cross-sectional descriptive study design and stratified cluster random sampling. According to 2010 data from the Tainan Bureau of Education, the total number of students in Tainan County was around 10,000 (the sampling frame). For stratification, we used geographic region (*n* =5) and city (*n* =31) with 41 public schools contributing to the sampling unit. In this study, a two-stage stratified sampling method was applied. First, we applied a proportionate allocation strategy to sample six middle schools and three high schools, which were selected randomly on the basis of the public schools. Next, all the school class numbers in the selected units were listed.

Participants were recruited from 28 classes of six middle schools and three high schools in Tainan County. The inclusion criteria were: (1) age between 13 and 19 years, (2) middle or high school student in Tainan County, (3) teacher’s report for each class that the student had no physical or mental handicap, (4) ability to complete self-administered questionnaires in Mandarin, and (5) informed consent of a parent or guardian prior to enrolment in the study. The determination of sample size was based on the need to perform factor analysis, which requires a minimum of 300 samples [22, 23]. Initially, 901 participants were invited to participate, and 87 either declined or did not complete the questionnaires. The resulting sample consisted of 814 participants.

### Procedure and ethical considerations

This study was approved by the Ethical Committee of the Institutional Review Board (Chang-Gung Memorial Hospital Ethics Committee No 99-1411B). Both participants and their parents (or guardians) gave their informed consent. Participants were notified about the survey by school health nurses and physical education teachers. Participants also had the opportunity to review the study questionnaire before indicating whether they wanted to participate. For ethical reasons, a cover letter was attached to each questionnaire, emphasizing that participants’ responses were anonymous and would remain confidential. Confidentiality was also maintained during data analysis by delinking questionnaire data from any personal identification information, such as name and birthday.

### Measurements

#### The original Adolescent Health Promotion (AHP) scale

The original AHP-40 is a self-administered instrument designed by Chen et al. [18] to measure adolescent health promoting behaviors. The instrument uses a 5-point Likert-type response format to obtain data regarding frequency of reported behaviors with scores ranging from 1 (*never*) to 5 (*always*). The total score ranges from 40 to 200, with higher scores indicating better health promoting behaviors. The original AHP-40 was developed in three steps and employed both qualitative and quantitative measures. In the first step, qualitative methods (in-depth interviews, focus groups with 14 experts in five areas, and a relevant literature review) were applied to generate the item pools of the AHP.

In the second step, quantitative methods were used to establish the reliability and validity of the AHP-40 items and subscales. Preliminary validity and reliability of the AHP-40 were established in 2003 using a sample of 1,128 adolescents [18]. Results indicated that the AHP-40 had good internal reliability and validity. Overall, the instrument had high internal consistency, with a Cronbach’s alpha coefficient of 0.93. Alpha coefficients for the six subscales ranged from 0.75 to 0.88 [18]. EFA was then used to establish the construct validity of the AHP-40, with results indicating that the AHP is comprised of six subscales: social support (7 items), life appreciation (8 items), health responsibility (8 items), nutritional behaviors (6 items), exercise behaviors (5 items), and stress management (6 items).

In the third step, a cross-sectional survey of 660 adolescents was conducted in which cross-validation and discriminant analysis were used to evaluate the construct and discriminant validity of the AHP scale for overweight and non-overweight adolescents [19]. For total scale scores, cluster analyses revealed two distinct patterns that were designated as *healthy* and *unhealthy* behaviors. Discriminant analysis indicated that this clustering had good discriminant validity; that is, non-overweight adolescents tended to be classified as healthy, whereas overweight adolescents tended to be classified as unhealthy.

#### Socio-demographic information

Demographic data were collected regarding age, gender, education level, living arrangement, body weight, height, body mass index (BMI), number of breakfasts eaten, and time spent watching television (TV) and playing computer games. Height and weight were measured by school health nurses, and BMI was calculated using the standard formula: weight (kg) divided by height (m^2^). BMI was then plotted according to age and sex-specific cutoff points to define the different body sizes of adolescents according to nationally accepted guidelines [3]. Each student was classified as either overweight or not overweight. For example, a 15-year-old boy with a BMI >23.1 would be defined as overweight, whereas a 15-year-old girl would be considered overweight with a BMI >22.7. Number of breakfasts eaten (per week) was defined as the number of meals eaten before 8:00 a.m. from Monday to Sunday. The time cutoff of 8:00 was selected after discussions with school administrators and takes into account schedules in most Taiwanese schools. Participants received instructions about how to record their breakfast meals; for example, “Irrespective of its content, please treat the meal as your breakfast if it is eaten before 8:00 a.m.” Students also responded to a self-administered, open-ended question: “Generally speaking, how many days do you eat breakfast each week (between Monday and Sunday)?”

Participants were also asked about the amount of weekday time they spent watching TV and playing computer games. We defined time spent playing on the computer as “except for school requirements (e.g., assignments) or searching for academic information, on weekdays I used the computer for playing games or chatting, in both home and cyber café settings”. We defined TV watching by the statement, “except for school requirements, the time between turning on and turning off the TV set on weekdays” [13].

### Statistical analysis

The validity of the AHP short form (AHP-SF) was tested using confirmatory factor analysis (CFA) and discriminant validity measures.

### Confirmatory factor analysis

The CFA was conducted using IBM SPSS AMOS 22.0 with a maximum likelihood estimate (MLE) and two-step samples consisting of calibration and validation [24]. The total sample (*N =*814) was randomly divided into two subgroups of equal size and homogeneity of baseline characteristics (i.e., age, gender, weight status, education level, number of breakfasts per week, and weekday time spent watching TV and playing computer games). The first sample (calibration sample) was used to validate and modify the factor structure developed in a previous study [18, 19]. The second sample (validation sample) was used to verify the stability of the factor structures developed from the calibration sample (by using cross-validation with a tight strategy) [25].

For the calibration sample, the analysis consisted of two steps that modified the factor structure of the AHP-40. First, the items with a factor loading greater than 0.50 were considered “practically significant” and were therefore retained [24]. Second, the items that were too highly correlated with other items were deleted by examining the modification index (MI) of the additional specification of error covariance. A large MI (i.e., >50) between two items indicated that these two items measured the same concept and, therefore, that one of the items should be deleted [26]. The CFA model was modified until all of the model fit indices met the established criteria. Specifically, all deletions and modifications were incorporated one by one and the CFA model was re-specified following each modification.

The goodness of fit of the model was assessed using absolute fit indices, relative fit indices and parsimony-based fit indices [24]. The absolute fit indices included the goodness of fit index (GFI), adjusted GFI index (AGFI), standardized root mean squared residual (SRMR), and root mean square error of approximation (RMSEA). The relative fit indices were assessed by the normed fit index (NFI), non-normed fit index (NNFI), and comparative fit index (CFI). Finally, the parsimony-based fit indices were assessed using the parsimony normed fit index (PNFI), parsimony comparative fit index (PCFI), and the likelihood-ratio (χ^2^/*df*). The model fit between the hypothesized model and observed data was considered “relatively good” if the following criteria were met: a cutoff value of GFI, AGFI, NFI, NNFI, and CFI greater than 0.90, a cutoff value of PNFI and PCFI greater than 0.50, a cutoff value of χ^2^/*df* lower than 3, and a cutoff value of SRMR and RMSEA less than 0.05 (good fit) or between 0.05 and 0.08 (adequate fit) [27].

The convergent validity of factor structures was established if the item’s factor loading was modest to high. Factor loadings higher than 0.50 were considered “practically significant” and considered a basic requirement of convergent validity. The internal consistency (reliability) of the factor structures was examined by both McDonald’s omega and Cronbach’s alpha coefficients for which a value greater than 0.70 would be considered acceptable [28].

### Discriminant validity

Discriminant validity was examined using logistic regression analysis on the whole sample to determine the association of the AHP-SF with three important health behaviors and health outcomes. The odds ratio (OR) was used as a measure of association.

## Results

### Characteristics of the participants

The majority of the respondents were Taiwanese students with ages ranging from 13 to 19 years. Of the 814 participants, 384 (47%) were males and 430 (53%) females. Most of these adolescents lived with their parents (75%), and the remainder lived either with a single parent or in a school dormitory. According to the BMI criteria considering gender and age, 23% (*n* =187) of participants were classified as overweight or obese. The majority of participants (70.6%, *n* =575) ate breakfast fewer than five times per week, and 27% (*n* =220) watched TV and played computer games (on weekdays) for more than four hours per day.

### CFA results for the calibration sample

**Figure 1:**
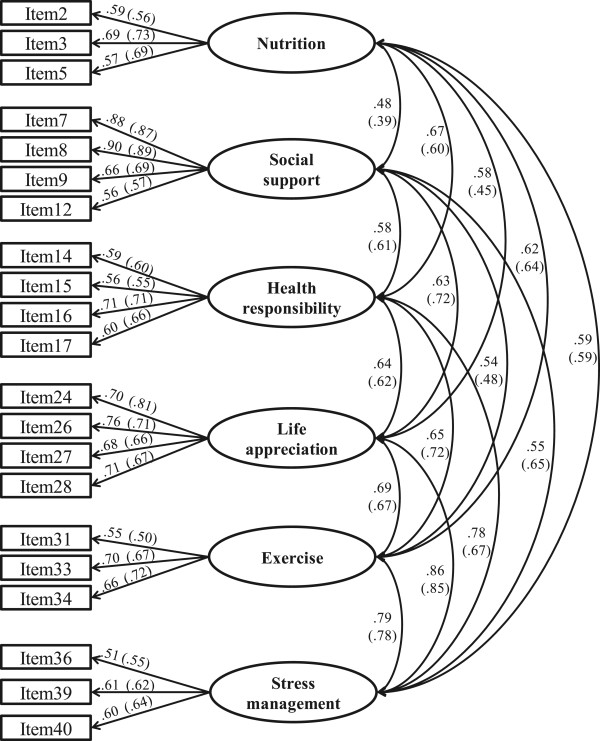
**The standardized estimates of CFA model for calibration and validation sample.** (The values of validation sample were in parenthesis).

As shown in Table 1, the initial 40-item CFA of the calibration sample revealed that half of the model fit indices failed to meet the established criteria, including GFI (0.81), AGFI (0.78), NFI (0.75), NNFI (0.82), and CFI (0.84). Three indices met the criteria, including PNFI (0.70), PCFI (0.78), and χ^2^/*df* (2.43). The SRMR (0.064) and RMSEA (0.059) were both between 0.05 and 0.08, which indicated an acceptable, fit. Generally, the initial 40-item model did not fit very well and thus needed modification. As a first step, 16 items were sequentially deleted (one by one) due to their low factor loadings (<0.50). However, after this 16-item deletion, the 24-item AHP short form still showed an inadequate relative model fit in terms of the NFI (0.88 which is less than the cutoff value of 0.90). In step two, we examined the modification indices of the 24 AHP items and found that three pairs of items appeared to measure the same three concepts (items 26 and 35, items 9 and 37, and items 20 and 40). After review by a panel of experts, items 35, 37, and 20 were deleted so that the final AHP-SF contained 21 items. For this set of items, all model fit indices met the criteria, suggesting a good model fit. Figure 1 shows the correlation matrices among the latent variables and factor loadings for the calibration and validation samples. As Figure 1 illustrates, all standardized factor loadings exceeded the threshold of 0.50, indicating an acceptable convergent validity of the AHP-SF [24]. In addition, McDonald’s omega for the AHP-SF was 0.904 (Cronbach’s alpha 0.905) indicating excellent internal consistency (data are not shown in the table).

**Table 1:** **Model fit indices of the proposed and modified CFA model for calibration and validation samples**

Global model fit	Calibration sample (40 items)	Calibration sample (24 items)	Calibration sample (21 items)	Validation sample (21 items)
Absolute fit index				
GFI	.81	.93	.95	.92
AGFI	.78	.91	.93	.90
SRMR	.064	.041	.036	.046
RMSEA	.059	.037	.028	.051
Relative fit index				
NFI	.75	.88	.93	.90
NNFI	.82	.95	.98	.93
CFI	.84	.96	.98	.94
Parsimony fit index				
PNFI	.70	.77	.77	.74
PCFI	.78	.82	.81	.78
LR χ^2^/*df*	2.43	1.56	1.31	2.07

*Note*: GFI = global fit index; AGFI = adjusted global fit index; SRMR = standardized root mean square residual; RMSEA = root mean square error of approximation; NFI = normed fit index; NNFI = non-normed fit index; CFI = comparative fit index; PNFI = parsimony normed fit index; PCFI = parsimony comparative fit index.

### CFA results for the validation sample and test of cross-validation

As illustrated in Table 1 for the validation sample, CFA of the 21-item AHP-SF showed that all indices of model fit met the established criteria. Although the validation sample showed a slightly worse model fit than the calibration sample, the sizes of the factor loadings were similar for the two samples (Table 2).

**Table 2:** **Factor loading, reliability and convergent validity of CFA for calibration and validation samples for the final 21-item CFA model**

	Calibration sample		Validation sample	
Construct/Item	***Mean***	***SD***	***Mean***	***SD***
**Nutrition**				
Item 2	2.94	0.86	2.97	0.95
Item 3	3.41	1.07	3.40	1.04
Item 5	3.49	1.10	3.44	1.10
**Social support**				
Item 7	3.45	1.10	3.28	1.13
Item 8	3.62	1.06	3.47	1.13
Item 9	3.58	1.15	3.53	1.23
Item 12	3.96	1.00	3.92	1.03
**Health responsibility**				
Item 14	3.60	1.27	3.62	1.27
Item 15	3.18	1.29	3.19	1.31
Item 16	2.73	1.18	2.64	1.22
Item 17	2.67	1.23	2.57	1.24
**Life appreciation**				
Item 24	3.69	1.11	3.67	1.14
Item 26	3.33	1.07	3.27	1.11
Item 27	3.92	1.08	3.86	1.08
Item 28	3.69	1.22	3.61	1.21
**Exercise**				
Item 31	3.57	1.28	3.47	1.26
Item 33	3.04	1.24	3.12	1.28
Item 34	2.83	1.05	2.86	1.07
**Stress management**				
Item 36	3.26	1.27	3.20	1.30
Item 39	3.17	1.18	3.20	1.24
Item 40	3.59	1.12	3.58	1.16

Table 3 shows the results of the cross-validation test in terms of subsamples. For each invariance comparison, increases in chi-square values (△χ^2^) were not statistically significant and decreases in CFI values were less than 0.01. These results suggest equality of factor loadings, factor variances and covariances, and residuals across subgroups [25, 29]. In other words, the factor structure modified and developed in the calibration sample was replicated in the validation sample, suggesting cross-sample stability of the AHP-SF.

**Table 3:** **Measurement invariance tests in terms of subsample (calibration and validation sample) for the final 21-item CFA model (** ***N*** **=814)**

Model and invariance level	^Δ^ ***df***	^Δ^χ^2^	***P***value	^Δ^NFI	^Δ^IFI	^Δ^RFI	^Δ^NNFI	^Δ^CFI
Assuming model unconstrained to be correct								
Unconstrained	–	–	–	–	–	–	–	–
Measurement weights	21	14.2	.862	.002	.002	-.004	-.004	.001
Structural covariance	36	34.9	.519	.006	.006	-.004	-.005	<.001
Measurement residual	57	57.3	.463	.009	.010	-.006	-.007	<.001
Assuming model measurement weights to be correct								
Structural covariance	15	20.8	.145	.003	.003	-.001	-.001	-.001
Measurement residual	36	43.2	.192	.007	.007	-.003	-.003	-.001
Assuming model structural covariance to be correct								
Measurement residuals	21	22.4	.377	.004	.004	-.002	-.002	<.001

Additional file 1: Table S1 represents the results of measurement invariance test in terms of gender. The results show equality of factor loadings and factor variances and covariance across genders. As to the equality of residual, increases in chi-square values (△χ^2^) were significant, while decreases in CFI values were less than 0.01, indicating that the equality between genders was sustained [25, 29].

### Discriminant validity

The results of discriminant validity for the whole sample are shown in Table 4. Previous studies have shown that skipping breakfast and spending more than four hours each day watching TV and playing computer games among adolescents are negatively associated with adolescent health status (e.g., obesity) and with health-related behaviors, including school achievement [12, 13, 30]. We therefore focused on three indicators of unhealthy lifestyle: (1) being overweight or obese, (2) frequently skipping breakfast, and (3) excessive time spent watching TV or playing computer games. Using body size as the criterion, lower scores for social support (OR =1.32, *p* =0.002), life appreciation (OR =1.24, *p* =0.018), exercise behaviors (OR =1.20, *p* =0.039), and total AHP-SF score (OR =1.35, *p* =0.016) were associated with a higher likelihood of being overweight or obese. Further, a decrease in several health promoting behaviors was associated with skipping breakfast: nutrition (OR =1.55, *p* <0.001), health responsibility (OR =1.21, *p* =0.031), exercise (OR =1.20, *p* =0.031), stress management behaviors (OR =1.22, *p* =0.028), and total AHP-SF score (OR =1.43, *p* =0.003). Spending more than four hours per day watching TV and playing computer games was associated with decreased scores on nutrition (OR =1.78, *p* <0.001), social support (OR =1.49, *p* <0.001), health responsibility (OR =1.57, *p* <0.001), life appreciation (OR =1.40, *p* <0.001), exercise (OR =1.59, *p* <0.001), stress management (OR =1.59, *p* <0.001), and total AHP-SF score (OR =2.23, *p* <0.001).

**Table 4:** **Relationship between outcomes of body sizes, skip breakfast, time spent on TV/PC and six dimensions of AHP-SF for the whole sample (** ***N*** **=814)**

	The absence or presence of outcomes		Per 1 unit decrease of health promoting behaviors with presence of outcomes	
Outcomes/AHP-SF subscales	Absence	Presence	OR (95% CI)	***P***
Overweight/obese (23.0%, 187/814)				
Nutrition	3.29 ± 0.79	3.22 ± 0.80	1.13 (0.92–1.38)	0.260
Social support	3.65 ± 0.88	3.42 ± 0.95	1.32 (1.11–1.58)	0.002
Health responsibility	3.04 ± 0.94	2.98 ± 0.86	1.07 (0.90–1.28)	0.459
Life appreciation	3.67 ± 0.89	3.49 ± 0.90	1.24 (1.04–1.49)	0.018
Exercise	3.18 ± 0.93	3.03 ± 0.92	1.20 (1.01–1.44)	0.039
Stress management	3.35 ± 0.91	3.26 ± 0.89	1.11 (0.93–1.33)	0.243
Total AHP-SF	3.38 ± 0.66	3.24 ± 0.68	1.35 (1.06–1.73)	0.016
Number of breakfast per week <5 (70.6%, 575/814)				
Nutrition	3.47 ± 0.81	3.20 ± 0.77	1.55 (1.27–1.90)	<0.001
Social support	3.67 ± 0.87	3.57 ± 0.91	1.13 (0.95–1.35)	0.155
Health responsibility	3.14 ± 0.93	2.98 ± 0.90	1.21 (1.02–1.43)	0.031
Life appreciation	3.72 ± 0.85	3.60 ± 0.91	1.17 (0.98–1.39)	0.085
Exercise	3.26 ± 0.91	3.10 ± 0.93	1.20 (1.02–1.43)	0.031
Stress management	3.44 ± 0.90	3.29 ± 0.90	1.22 (1.02–1.45)	0.028
Total AHP-SF	3.46 ± 0.66	3.30 ± 0.66	1.43 (1.13–1.81)	0.003
TV and playing computer games ≧4 hours per day (27.0%, 220/814)				
Nutrition	3.37 ± 0.77	3.02 ± 0.79	1.78 (1.45–2.19)	<0.001
Social support	3.69 ± 0.86	3.37 ± 0.96	1.49 (1.25–1.77)	<0.001
Health responsibility	3.13 ± 0.89	2.76 ± 0.94	1.57 (1.32–1.88)	<0.001
Life appreciation	3.71 ± 0.88	3.44 ± 0.91	1.40 (1.18–1.67)	<0.001
Exercise	3.26 ± 0.89	2.86 ± 0.98	1.59 (1.34–1.90)	<0.001
Stress management	3.44 ± 0.87	3.06 ± 0.94	1.59 (1.34–1.90)	<0.001
Total AHP-SF	3.44 ± 0.64	3.10 ± 0.68	2.23 (1.74–2.85)	<0.001

AHP-SF = Adolescent Health Promotion short form; OR = odds ratio; CI = confidence interval.

## Discussion

The results of the present study demonstrate that the AHP-SF is a reliable and valid instrument for measuring adolescent health promoting behaviors. To our knowledge, this is the first study reporting the development and psychometric testing of such a parsimonious instrument for this population. Key strengths of the present study are that we included a large sample (*N* =814) of adolescents and that we conducted a strict two-sample analysis (using well-defined calibration and validation samples). Furthermore, we used multiple methods to establish the validity and reliability of the AHP-SF, including construct validity, convergent validity, discriminant validity, and internal consistency (reliability).

In addition to its strengths, the present study had some potential limitations. First, our study focused on the concept of health promotion without including a health protection dimension, such as promoting avoidance of alcohol consumption or smoking. Therefore, the AHP-SF is not a useful tool for assessing or identifying risk behaviors among youth. Second, procedures of test-retest reliability were not applied in this study, which are important for the assessment of an instrument’s stability. Further investigations need to assess the stability of the AHP-SF to avoid the instrument’s susceptibility to extraneous influences at different times. Third, our study sample was recruited only from public schools in one Taiwanese county. Therefore, our findings cannot be generalized to larger geographic areas or different countries and cultures. Future research will be required to confirm generalizability of our findings.

Despite these limitations, the present study confirmed that the AHP-SF provides a quick, practical, and comprehensive screening tool for health promotion behaviors in adolescents. Consequently, the instrument may be useful for early detection and modification of unhealthy lifestyle choices among adolescents in school settings. In addition, our study supports the previous finding that health promotion is a multidimensional concept [1, 31]. In particular, the AHP-SF consists of six distinct domains (Table 5): nutrition (3 items), social support (4 items), health responsibility (4 items), life appreciation (4 items), exercise (4 items), and stress management (3 items).

**Table 5:** **Adolescent health promotion short form (AHP-SF)**

Nutrition	21. Make an effort to choose foods without preservatives (e.g., additives on food).^a^
1. I eat three meals daily.^a^	Life appreciation
2. I choose foods without too much oil.	22. Make an effort to like myself.^a^
3. Include dietary fiber (e.g. fruits or vegetables).	23. Make an effort to feel happy and content.^a^
4. Drink at least 1500 cc of water daily (or 6–8 cups).^a^	24. I usually think positively.
5. Each meal includes five food groups (e.g. bread, meat, milk, fruit, vegetable)	25. Make an effort to understand my strengths, weaknesses and accept them.^a^
6. Eat breakfast daily.^a^	26. Make an attempt to correct my defects.
Social support	27. Make an effort to know what’s important for me.
7. I speak up & share my feelings with others.	28. Make an effort to feel interesting and challenged every day.
8. I care about other people.	29. Make an effort to believe that my life has purpose.^a^
9. I talk about my concerns with others.	Exercise
10. Make an effort to smile or laugh every day.^a^	30. Perform stretching exercise daily.^a^
11. Enjoy keeping in touch with relatives.^a^	31. Exercise rigorously 30 minutes at least 3 times per week.
12. Make an effort to have good friendships.	32. Participate in physical fitness class at school weekly.^a^
13. Talk about my troubles to others.^a^	33. Warm up before rigorous exercise.
Health responsibility	34. Make an effort to stand or sit up straight.
14. Read food labels when I shop.	Stress management
15. I watch my weight.	35. Make an effort to spend time daily for relaxation.^a^
16. Discuss my health concerns with a doctor or nurse.	36. Make an effort to determine the source of my stress.
17. Observe my body at least monthly.	37. Make an effort to watch my mood changes.^a^
18. Brush my teeth and use dental floss after meals.^a^	38. Sleep for 6–8 hours each night.^a^
19. Wash hands before meals.^a^	39. Make schedules and set priorities.
20. Read health information.^a^	40. I try not to lose control when things happen that are unfair.

^a^Items to be removed based on the CFA.

The World Health Organization points out that 60% of the quality of an individual’s health and life depends on his/her behavior and lifestyle [1]. The present study also indicates that the AHP-SF is closely related to current and subsequent health status. However, lifestyle changes can be difficult to achieve when adolescents spend a large part of their time in school, and teachers probably providing more adult contact than parents. Additionally, there is an abundance of research indicating that in modern, technological societies (in both developing and developed countries), many adolescents are addicted to screen-related devices and may adopt poor eating habits, do little exercise, build few interpersonal relationships, and have inadequate stress management skills [1, 3]. Therefore, the role of schools may need to include providing adolescents with accurate and meaningful health information as well as the skills they need to make informed, deliberate, and constructive decisions in their lives. Healthy life-skills training can be a vital part of this decision-making process. A reliable and valid instrument, such as the AHP-SF, is important because measurement of health-promotion related behaviors is essential to identify the health-related lifestyle needs of individual adolescents and to evaluate the outcome of health promotion programs.

## Conclusion

The present study shows that the 21-item AHP-SF is a short, valid, and reliable instrument for evaluating health-related lifestyle choices for adolescents in Taiwan. This conclusion is based on multiple acceptable validity tests and several internal consistency estimates. The factor structure results of the CFA for the calibration sample were mostly replicated in the validation sample, suggesting that the AHP-SF has cross-sample stability. Because of the importance of health promotion in improving youth health, reliable and valid instruments are required for measuring health-promoting behaviors. The AHP-SF fulfils this requirement by providing a practical tool for assessing adolescent health-related behaviors; this tool may be used by primary health care providers, school nurses, and community health centers. It may also be helpful for researchers who are attempting to develop intervention programs related to improving adolescent health. In addition, the AHP-SF could be used as an assessment and evaluation tool in school health centers for daily health counseling.

## Electronic supplementary material

Additional file 1: Table S1: Measurement invariance tests in terms of gender for the final 21-item CFA model (*N* = 814). (DOC 34 KB)
