# Supplementary material for: Development and validation of the short-form adolescent health promotion scale
Source: BMC Public Health. 2014 Oct 26;14:1106. doi: 10.1186/1471-2458-14-1106 (PMC4216378; doi:10.1186/1471-2458-14-1106)
Supplement: Supplementary file 1 — Additional file 1: Table S1: Measurement invariance tests in terms of gender for the final 21-item CFA model (N = 814). (DOC 34 KB) [file 12889_2014_7192_MOESM1_ESM.doc]

Supplement Table 1. Measurement invariance tests in terms of gender for the final 21-item CFA model (*N* = 814)

| Model and invariance level | △*df* | △χ^2^ | *P* value | △NFI | △IFI | △RFI | △NNFI | △CFI |
| --- | --- | --- | --- | --- | --- | --- | --- | --- |
| Assuming model unconstrained to be correct | | | | | | | | |
| Unconstrained | – | – | – | – | – | – | – | – |
| Measurement weights | 21 | 10.09 | .978 | .002 | .002 | -.005 | -.005 | .002 |
| Structural covariance | 36 | 34.39 | .545 | .005 | .006 | -.005 | -.005 | .001 |
| Measurement residual | 57 | 94.46^**^ | .001 | .015 | .016 | <.001 | <.001 | -.006 |
| Assuming model measurement weights to be correct | | | | | | | | |
| Structural covariance | 15 | 24.30 | .060 | .004 | .004 | <.001 | <.001 | .001 |
| Measurement residual | 36 | 84.37^***^ | <.001 | .013 | .014 | .004 | .005 | .008 |
| Assuming model structural covariance to be correct | | | | | | | | |
| Measurement residuals | 21 | 60.07^***^ | <.001 | .010 | .010 | .004 | .005 | -.007 |

****p* < .05.
